# Supplementary material for: Comparative Effectiveness and Safety of Concomitant Treatment with Chuna Manual Therapy and Usual Care for Whiplash Injuries: A Multicenter Randomized Controlled Trial
Source: Int J Environ Res Public Health. 2022 Aug 27;19(17):10678. doi: 10.3390/ijerph191710678 (PMC9518174; doi:10.3390/ijerph191710678)
Supplement: Supplementary file 1 [file ijerph-19-10678-s001.zip › tableS1_.pdf]

Supplemental Table S1. Clinical study schedule for the treatment of patients with whiplash injury

**Period**	**Screening**	**Active Treatment**			**Follow up**			
	**Week 0**	**Week 1 (D0)**	**Week 2**	**Week 3**	**Week 4 (D+21)**	**Week 7 (D+42)**	**Week 13 (D+84)**	**Week 25 (D+168)**
Measure	Visit 1 Screening	Visit 2 (First Visit)		(Visit after 2 Weeks)	F/u 1 (Visit after 3 Weeks)	F/u 2 (Visit after 6 Weeks)	F/u 3 (Visit after 12 Weeks)	F/u 4 (Visit after 24 Weeks)
Participant Consent Form	○							
Vital sign	○							
Information of Demographic and Sociological characteristics[a]	○							
Information of Traffic Accident[b]	○							

Confirm Suitability	○							
Randomization		○						
Credibility and Expectancy		○						
Intervention<br>UC or CMT+UC		2–5 times	2–3 times	2–3 times				
NRS	○	○	○	○	○	○	○	○
NDI		○			○	○	○	
SF-12		○			○	○	○	
PGIC					○	○	○	
Adverse Event		every visit	every visit	every visit	○	○	○	○
Concomitant Drug		○	○	○	○	○	○	

[a] Age, gender, occupation, body index, past history, drug history, body mass index

[b] Date of accident, symptoms, diagnosis

CMT, Chuna manual therapy; D, day; f/u, follow-up; NDI, Neck Disability Index; NRS, numeric rating scale; PGIC, Patient's Global Impression of Change;

SF-12 (MCS) 12-item short-form health survey mental component summary; SF-12 (PCS), 12-item short-form health survey physical component summary;

UC, usual care
