# Supplementary material for: Comparative Effectiveness and Safety of Concomitant Treatment with Chuna Manual Therapy and Usual Care for Whiplash Injuries: A Multicenter Randomized Controlled Trial
Source: Int J Environ Res Public Health. 2022 Aug 27;19(17):10678. doi: 10.3390/ijerph191710678 (PMC9518174; doi:10.3390/ijerph191710678)
Supplement: Supplementary file 1 [file ijerph-19-10678-s001.zip › tableS2_.pdf]

Supplemental Table S2. Medically related adverse events after treatment of whiplash injury

Symptom/disease	CMT + UC (n = 66) n (%)	UC alone (n = 66) n (%)
Headache	1 (1.5)	1 (1.5)
Fibromyalgia, Dizziness, Nausea	1 (1.5)	0
Itching	0	1 (1.5)
Paresthesia	0	1 (1.5)
Total	2 (3)	3 (4.5)

CMT, Chuna manual therapy; UC, usual care
