# Supplementary material for: Comparative Effectiveness and Safety of Concomitant Treatment with Chuna Manual Therapy and Usual Care for Whiplash Injuries: A Multicenter Randomized Controlled Trial
Source: Int J Environ Res Public Health. 2022 Aug 27;19(17):10678. doi: 10.3390/ijerph191710678 (PMC9518174; doi:10.3390/ijerph191710678)
Supplement: Supplementary file 1 [file ijerph-19-10678-s001.zip › tableS3_.pdf]

**Supplemental Table S3. Per-protocol analysis of primary and secondary outcomes of whiplash injury treatment**

| Variable | Observed value | | | Change from baseline | | | | |
|---|---|---|---|---|---|---|---|---|
| | UC alone (n = 60) | CMT+UC (n = 61) | P-value[b] | UC alone (n = 60) | P-value[a] | CMT+UC (n = 61) | P-value[a] | P-value[b] |
| **50% pain reduction day** | | | | | | | | |
| F/U 4 | 51.36 ± 48.43 | 23.31 ± 21.36 | **.001[d]** | - | - | - | | |
| **NRS_AUC** | | | | | | | | |
| F/U 4 | 685.19 ± 309.86 | 468.31 ± 285.01 | **<.001[d]** | | | | | |
| **NRS** | | | | | | | | |
| visit 2 | 6.35 ± 1.04[a] | 6.36 ± 1.10[a] | .956[d] | | | | | |
| F/U 1 | 5.00 ± 1.90[b] | 3.80 ± 1.98[b] | **.001[d]** | -1.35 ± 1.49 | **<.001** | -2.56 ± 1.98 | **<.001** | **<.001[d]** |
| F/U 2 | 4.60 ± 1.89[b] | 3.10 ± 1.98[c] | **<.001[d]** | -1.75 ± 1.55 | **<.001** | -3.26 ± 1.92 | **<.001** | **<.001[d]** |
| F/U 3 | 3.87 ± 2.34[c] | 2.02 ± 1.89[d] | **<.001[d]** | -2.48 ± 1.85 | **<.001** | -4.34 ± 2.03 | **<.001** | **<.001[d]** |

| Variable | Observed value | | | Change from baseline | | | | |
|---|---|---|---|---|---|---|---|---|
| | UC alone (n = 60) | CMT+UC (n = 61) | P-value[b] | UC alone (n = 60) | P-value[a] | CMT+UC (n = 61) | P-value[a] | P-value[b] |
| F/U 4 | 3.02 ± 2.41[d] | 2.18 ± 2.33[d] | .055[d] | -3.33 ± 2.15 | **<.001** | -4.18 ± 2.43 | **<.001** | **.045[d]** |
| **P value[c]** | **<.001[f]** | **<.001[f]** | | | | | | |
| **NDI** | | | | | | | | |
| visit 2 | 20.62 ± 6.75[a] | 20.10 ± 6.66[a] | .671[d] | | | | | |
| F/U 1 | 15.38 ± 7.21[b] | 12.80 ± 6.47[b] | **.040[d]** | -5.23 ± 4.59 | **<.001** | -7.30 ± 5.47 | **<.001** | **.027[d]** |
| F/U 2 | 13.52 ± 6.39[c] | 10.41 ± 5.65[c] | **.005[d]** | -7.10 ± 5.49 | **<.001** | -9.69 ± 6.34 | **<.001** | **.018[d]** |
| F/U 3 | 11.75 ± 7.73[c] | 8.33 ± 5.97[d] | **.007[d]** | -8.87 ± 7.37 | **<.001** | -11.77 ± 6.93 | **<.001** | **.027[d]** |
| **P value[c]** | **<.001[f]** | **<.001[f]** | | | | | | |
| **PGIC** | | | | | | | | |
| F/U 1 | 3.0 (1.0)[a] | 2.0 (1.0)[a] | **.003[e]** | | | | | |
| F/U 2 | 3.0 (1.0)[a] | 2.0 (1.0)[b] | **<.001[e]** | - | - | - | - | - |

| Variable | Observed value | | | Change from baseline | | | | |
|---|---|---|---|---|---|---|---|---|
| | UC alone (n = 60) | CMT+UC (n = 61) | P-value[b] | UC alone (n = 60) | P-value[a] | CMT+UC (n = 61) | P-value[a] | P-value[b] |
| F/U 3 | 2.0 (1.0)[b] | 2.0 (1.0)[c] | **.001[e]** | - | - | - | - | - |
| **P value[c]** | **<.001[g]** | **<.001[g]** | | | | | | |
| **Credibility & Expectancy** | | | | | | | | |
| visit 2 | 7.0 (2.0) | 7.0 (3.0) | .432[e] | | | | | |
| **SF-12(PCS)** | | | | | | | | |
| visit 2 | 38.84 ± 7.29[a] | 39.78 ± 7.15[a] | .478[d] | | | | | |
| F/U 1 | 42.88 ± 7.52[b] | 44.93 ± 5.79[b] | .094[d] | 4.03 ± 7.26 | **<.001** | 5.15 ± 7.04 | **<.001** | .389[d] |
| F/U 2 | 44.02 ± 6.70[b] | 46.79 ± 6.78[b] | **.026[d]** | 5.18 ± 8.35 | **<.001** | 7.01 ± 7.65 | **<.001** | .211[d] |
| F/U 3 | 46.44 ± 7.30[c] | 49.20 ± 6.90[c] | **.035[d]** | 7.59 ± 9.82 | **<.001** | 9.42 ± 8.19 | **<.001** | .268[d] |
| **P value[c]** | **<.001[f]** | **<.001[f]** | | | | | | |

| Variable | Observed value | | | Change from baseline | | | | |
|---|---|---|---|---|---|---|---|---|
| | UC alone (n = 60) | CMT+UC (n = 61) | P-value[b] | UC alone (n = 60) | P-value[a] | CMT+UC (n = 61) | P-value[a] | P-value[b] |
| **SF-12(MCS)** | | | | | | | | |
| visit 2 | 38.88 ± 11.10[a] | 41.04 ± 10.17[a] | .269[d] | | | | | |
| F/U 1 | 44.95 ± 9.68[b] | 47.35 ± 9.83[b] | .178[d] | 6.07 ± 9.26 | **<.001** | 6.32 ± 8.22 | **<.001** | .876[d] |
| F/U 2 | 46.77 ± 10.69[bc] | 51.72 ± 9.31[c] | **.007[d]** | 7.88 ± 8.46 | **<.001** | 10.69 ± 8.93 | **<.001** | .079[d] |
| F/U 3 | 49.47 ± 9.87[c] | 52.42 ± 8.96[c] | .088[d] | 10.59 ± 9.94 | **<.001** | 11.38 ± 9.72 | **<.001** | .657[d] |
| **P value[c]** | **<.001[f]** | **<.001[f]** | | | | | | |

[a] P-values were derived from a paired t-test for within-group comparisons.

[b] P-values were derived from between-group comparisons.

[c] P-values were derived from comparing changes over time.

[d] P-values were derived from an independent t-test.

[e] P-values were derived from the Mann–Whitney U test.

[f] P-values were derived from RM-ANOVA.

[g] P-values were derived from the Friedman test.

AUC, area under the Receiver Operating Characteristic Curve; CMT, Chuna manual therapy; NDI, Neck Disability Index; NRS, numeric rating scale; PGIC, Patient's Global Impression of Change; SF-12 (MCS) 12-item short-form health survey mental component summary; SF-12 (PCS), 12-item short-form health survey physical component summary; UC, usual care
