# Supplementary material for: Comparative Effectiveness and Safety of Concomitant Treatment with Chuna Manual Therapy and Usual Care for Whiplash Injuries: A Multicenter Randomized Controlled Trial
Source: Int J Environ Res Public Health. 2022 Aug 27;19(17):10678. doi: 10.3390/ijerph191710678 (PMC9518174; doi:10.3390/ijerph191710678)
Supplement: Supplementary file 1 [file ijerph-19-10678-s001.zip › tableS4_.pdf]

Supplemental Table S4. Subgroup analysis based on the severity of WAD 1 and 2

4-1. Patient baseline characteristics (Subgroup)

**Variable**		**WAD1 (n = 71)**	**WAD2 (n = 61)**	**P-value**	**WAD 1**			**WAD 2**		
					**WAD1 UC alone (n = 37)**	**WAD1 CMT +U C (n = 34)**	**P-value**	**WAD2 UC alone (n = 29)**	**WAD2 CMT + UC (n = 32)**	**P-value**
**Sex**	Male	14 (19.7%)	27 (44.3%)	**.002**[a]	9 (24.3%)	5 (14.7%)	.309[a]	15 (51.7%)	12 (37.5%)	.264[a]
	Female	57 (80.3%)	34 (55.7%)		28 (75.7%)	29 (85.3%)		14 (48.3%)	20 (62.5%)	
**Age (years)**		43.08 ± 12.54	36.31 ± 9.77	**.001**[b]	44.05 ± 12.47	42.03 ± 12.72	.501[b]	35.52 ± 9.96	37.03 ± 9.70	.431[3)]
**Height (cm)**		163.85 ± 7.88	167.67 ± 7.88	**.006**[b]	164.32 ± 8.67	163.33 ± 7.02	.601[b]	168.19 ± 7.62	167.19 ± 8.20	.398[3)]
**Weight (kg)**		62.40 ± 11.93	66.67 ± 13.06	.052[b]	63.92 ± 11.79	60.75 ± 12.04	.266[b]	68.92 ± 13.96	64.63 ± 12.04	.181[3)]
**BMI**		23.37 ± 3.77	23.65 ±	.663[b]	23.92 ± 3.20	22.77 ± 4.27	.203[b]	24.38 ± 4.35	22.99 ± 2.67	.146[b]

	3.61					

[a] P-values were derived from the chi-square test.

[b] P-values were derived from an independent t-test for between-group comparisons.

BMI, body mass index; CMT, Chuna manual treatment; UC, usual care; WAD, whiplash-associated disorder.

4-2 Subgroup analysis of primary and secondary outcomes of whiplash injury treatment

Variable	WAD1			WAD2		
	UC alone (n = 37)	CMT + UC (n = 34)	P-value[b]	UC alone (n = 29)	CMT + UC (n = 32)	P-value[b]
**NRS_D-day**						
F/U 4	53.89 ± 50.69	16.37 ± 9.37	**.001[d]**	45.47 ± 45.61	31.64 ± 28.06	.586[e]
**NRS_AUC**						
F/U 4	676.06 ± 294.48	416.43 ± 235.98	**<.001[d]**	685.54 ± 307.40	547.06 ± 304.37	.083[d]
**NRS**						

Variable	WAD1			WAD2		
	UC alone (n = 37)	CMT + UC (n = 34)	P-value[b]	UC alone (n = 29)	CMT + UC (n = 32)	P-value[b]
visit 2	6.38 ± 1.04[a]	6.24 ± 0.96[a]	.548[d]	6.28 ± 1.00[a]	6.44 ± 1.22[a]	.649[e]
F/U 1	5.08 ± 1.67[b]	3.18 ± 1.62[b]	**<.001[d]**	4.83 ± 2.00[b]	4.59 ± 1.97[b]	.647[d]
F/U 2	4.89 ± 1.76[b]	2.62 ± 1.67[bc]	**<.001[d]**	4.24 ± 1.86[b]	3.75 ± 2.03[c]	.331[d]
F/U 3	3.73 ± 2.26[c]	1.56 ± 1.54[d]	**<.001[d]**	3.90 ± 2.29[bc]	2.66 ± 1.98[d]	**.030[e]**
F/U 4	2.84 ± 2.17[d]	2.26 ± 2.09[cd]	.262[d]	3.17 ± 2.54[c]	2.28 ± 2.52[d]	.134[e]
**P value[c]**	**<.001[f]**	**<.001[f]**		**<.001[6)]**	**<.001[6)]**	
**NDI**						
visit 2	20.78 ± 6.71[a]	20.15 ± 6.16[a]	.679[d]	19.55 ± 7.39[a]	19.56 ± 7.61[a]	.996[d]
F/U 1	14.78 ± 7.28[b]	12.41 ± 5.89[b]	.138[d]	15.24 ± 7.32[b]	13.56 ± 6.93[b]	.298[e]
F/U 2	13.54 ± 5.07[b]	9.74 ± 5.72[c]	**.004[d]**	13.21 ± 7.41[bc]	11.63 ± 5.30[b]	.613[e]
F/U 3	11.57 ± 8.08[b]	7.76 ± 6.21[d]	**.030[d]**	11.72 ± 6.80[c]	9.50 ± 5.45[c]	.162[d]

Variable	WAD1			WAD2		
	UC alone (n = 37)	CMT + UC (n = 34)	P-value[b]	UC alone (n = 29)	CMT + UC (n = 32)	P-value[b]
**P value[c]**	**<.001[f]**	**<.001[f]**		**<.001[6)]**	**<.001[6)]**	
**PGIC**						
F/U 1	3.0 (1.0)[a]	2.0 (0.0)[a]	**.001[e]**	3.0 (1.0)[ab]	3.0 (1.0)[a]	.360[e]
F/U 2	3.0 (1.0)[ab]	2.0 (1.0)[ab]	**<.001[e]**	3.0 (1.0)[a]	2.0 (1.0)[a]	.150[e]
F/U 3	2.0 (1.0)[b]	2.0 (1.0)[b]	**.002[e]**	2.0 (1.0)[b]	2.0 (2.0)[b]	.118[e]
**P value[c]**	**.001[g]**	**.004[g]**		**.023[6)]**	**.001[6)]**	
**Credibility & Expectancy**						
visit 2	7.0 (2.0)	8.0 (2.0)	.063[e]	6.0 (2.5)	6.0 (1.5)	.836[e]
**SF-12(PCS)**						
visit 2	38.93 ± 7.13[a]	38.64 ± 6.50[a]	.863[d]	39.65 ± 7.16[a]	41.65 ± 7.65[a]	.297[d]

Variable	WAD1			WAD2		
	UC alone (n = 37)	CMT + UC (n = 34)	P-value[b]	UC alone (n = 29)	CMT + UC (n = 32)	P-value[b]
F/U 1	42.46 ± 8.24[b]	45.28 ± 5.19[b]	.087[d]	44.02 ± 6.27[b]	44.24 ± 6.08[a]	.893[d]
F/U 2	43.36 ± 6.64[b]	46.35 ± 7.28[b]	.075[d]	45.09 ± 6.45[b]	46.85 ± 5.95[b]	.271[d]
F/U 3	46.71 ± 6.99[c]	48.73 ± 7.29[c]	.238[d]	46.44 ± 7.50[b]	49.13 ± 6.18[b]	.131[d]
**P value[c]**	**<.001[f]**	**<.001[f]**		**<.001[5)]**	**<.001[5)]**	
**SF-12(MCS)**						
visit 2	39.05 ± 10.27[a]	40.66 ± 10.15[a]	.510[d]	38.73 ± 11.89[a]	41.43 ± 10.32[a]	.346[d]
F/U 1	46.03 ± 9.57[b]	47.89 ± 9.45[b]	.412[d]	43.79 ± 9.21[ab]	46.37 ± 10.17[b]	.341[e]
F/U 2	47.20 ± 10.30[b]	52.09 ± 9.91[c]	**.046[d]**	46.32 ± 10.81[bc]	51.22 ± 8.77[c]	.091[e]
F/U 3	49.28 ± 10.67[b]	52.19 ± 9.96[c]	.241[d]	49.62 ± 8.25[c]	52.25 ± 8.12[c]	.175[e]
**P value[c]**	**<.001[f]**	**<.001[f]**		**<.001[6)]**	**<.001[6)]**	

[a] P-values were derived from a paired t-test for within-group comparisons.

[b] P-values were derived from between-group comparisons.

[c] P-values were derived from comparing changes over time.

[d] P-values were derived from an independent t-test.

[e] P-values were derived from the Mann–Whitney U test.

[f] P-values were derived from RM-ANOVA.

[g] P-values were derived from the Friedman test.

AUC, area under the receiver operating characteristic curve; CMT, Chuna manual therapy; f/u, follow-up; f/u1, post-intervention week 4; f/u2, post-intervention week 7; f/u3, post-intervention week 13; f/u4, post-intervention week 25; NDI, Neck Disability Index; NRS, numeric rating scale; PGIC, Patient's Global Impression of Change; RM-ANOVA, repeated measurement analysis of variance; SF-12 (MCS) 12-item short-form health survey mental component summary; SF-12 (PCS), 12-item short-form health survey physical component summary; UC, usual care; WAD, whiplash-associated disorder
